# Accuracy of ultra-high resolution and virtual non-calcium reconstruction algorithm for stenosis evaluation with photon-counting CT: results from a dynamic phantom study

**DOI:** 10.1186/s41747-024-00482-w

**Published:** 2024-08-29

**Authors:** Emese Zsarnoczay, Nicola Fink, U. Joseph Schoepf, Daniel Pinos, Jim O’Doherty, Thomas Allmendinger, Junia Hagenauer, Joseph P. Griffith III, Milán Vecsey-Nagy, Pál Maurovich-Horvat, Tilman Emrich, Akos Varga-Szemes

**Affiliations:** 1 Division of Cardiovascular Imaging, Department of Radiology and Radiological Science, Medical University of South Carolina, Charleston, SC USA; 2 MTA-SE Cardiovascular Imaging Research Group, Medical Imaging Centre, Semmelweis University, Budapest, Hungary; 3 Department of Radiology, University Hospital, LMU Munich, Munich, Germany; 4 Siemens Medical Solutions USA Inc, Malvern, PA USA; 5 Siemens Healthcare GmbH, Forchheim, Germany; 6 Faculty of Medicine, Friedrich Alexander University of Erlangen-Nuremberg, Erlangen, Germany; 7 Cardiovascular Imaging Research Group, Heart and Vascular Center, Semmelweis University, Budapest, Hungary; 8 Department of Diagnostic and Interventional Radiology, University Medical Center of the Johannes Gutenberg-University, Mainz, Germany; 9 German Centre for Cardiovascular Research, Partner site Rhine-Main, Mainz, Germany

**Keywords:** Computed tomography angiography, Coronary stenosis, Heart rate, Phantoms (imaging), Tomography (x-ray computed)

## Abstract

**Background:**

We compared ultra-high resolution (UHR), standard resolution (SR), and virtual non-calcium (VNCa) reconstruction for coronary artery stenosis evaluation using photon-counting computed tomography (PC-CT).

**Methods:**

One vessel phantom (4-mm diameter) containing solid calcified lesions with 25% and 50% stenoses inside a thorax phantom with motion simulation underwent PC-CT using UHR (0.2-mm slice thickness) and SR (0.6-mm slice thickness) at heart rates of 60 beats per minute (bpm), 80 bpm, and 100 bpm. A paired *t*-test or Wilcoxon test with Bonferroni correction was used.

**Results:**

For 50% stenosis, differences in percent mean diameter stenosis between UHR and SR at 60 bpm (51.0 vs 60.3), 80 bpm (51.7 vs 59.6), and 100 bpm (53.7 vs 59.0) (*p* ≤ 0.011), as well as between VNCa and SR at 60 bpm (50.6 vs 60.3), 80 bpm (51.5 vs 59.6), and 100 bpm (53.7 vs 59.0) were significant (*p* ≤ 0.011), while differences between UHR and VNCa at all heart rates (*p* ≥ 0.327) were not significant. For 25% stenosis, differences between UHR and SR at 60 bpm (28.0 vs 33.7), 80 bpm (28.4 vs 34.3), and VNCa vs SR at 60 bpm (29.1 vs 33.7) were significant (*p* ≤ 0.015), while differences for UHR vs SR at 100 bpm (29.9 vs 34.0), as well as for VNCa vs SR at 80 bpm (30.7 vs 34.3) and 100 bpm (33.1 vs 34.0) were not significant (*p* ≥ 0.028).

**Conclusion:**

Stenosis quantification accuracy with PC-CT improved using either UHR acquisition or VNCa reconstruction.

**Relevance statement:**

PC-CT offers to scan with UHR mode and the reconstruction of VNCa images both of them could provide improved coronary stenosis quantification at increased heart rates, allowing a more accurate stenosis grading at low and high heart rates compared to SR.

**Key Points:**

- Evaluation of coronary stenosis with conventional CT is challenging at high heart rates.
- PC-CT allows for scanning with ECG-gated UHR and SR modes.
- UHR and VNCa images were compared in a dynamic phantom.
- UHR improves stenosis quantification up to 100 bpm.
- VNCa reconstruction improves stenosis evaluation up to 80 bpm.

**Graphical Abstract:**

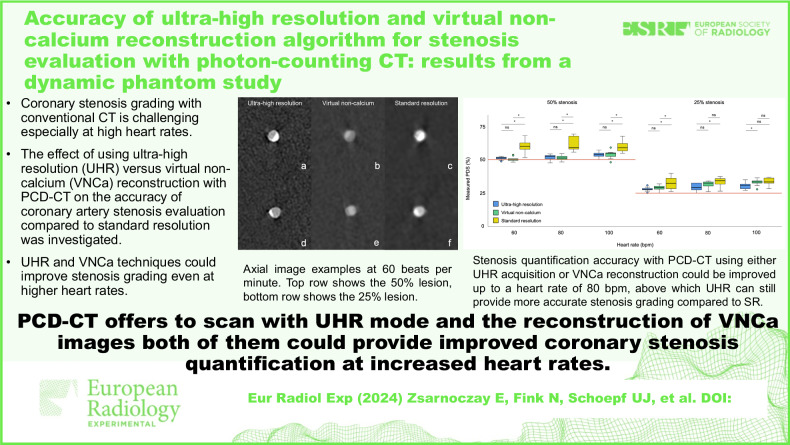

## Background

Coronary computed tomography (CT) angiography has emerged as an established noninvasive imaging modality with a class I indication for the evaluation of symptomatic patients with suspected coronary artery disease [1–3]. Extensive evidence has supported its effective role as a gatekeeper to invasive cardiac catheterization, which carries the risk of major complications [4]. However, limitations do exist. For example, the evaluation of the coronary lumen and stenosis severity may be challenging with conventional CT technology, especially in the presence of severe calcifications and/or at higher heart rates [5, 6]. High coronary calcification burden often leads to blooming artifacts causing an overestimation of stenosis grade [7]. As a consequence, the probability of a false-positive diagnosis and the overestimation of stenosis relevance are considerable with conventional techniques [8, 9].

The recently introduced whole-body, dual-source, photon-counting computed tomography (PC-CT) detector uses cadmium telluride crystal semiconductors that directly convert x-ray photons to electronic signals instead of an indirect conversion used by conventional systems [10]. PC detectors count every incident photon equally and determine their associated energy. Furthermore, electronic noise is reduced since only x-rays with an energy over 20 keV are counted by readout electronics [11]. Acquisition at ultra-high resolution (UHR) with conventional CT systems is limited by the detector pixel size which could not be reduced significantly in recent years. In conventional systems, spatial resolution can be improved by other techniques such as with the use of comb or grid filters, which however reduce radiation dose efficiency. On the other hand, PC-CT allows for UHR scanning with a specific scan mode utilizing a minimum detector pixel size of 0.151 × 0.176 mm^2^ at the isocenter while maintaining a high temporal resolution [12, 13]. PC-CT demonstrated the potential to reduce image artifacts and noise, while still improving dose efficiency and contrast-to-noise ratio [14–16].

In addition, the multi-energy capabilities of the PC-CT system make it possible to reconstruct spectral images scanned with standard resolution (SR) (collimation of 144 × 0.4 mm). Material decomposition and generation of spectrally postprocessed images, e.g., iodine removal with preserved calcification [17, 18] and calcium removal to visualize coronary artery lumen by separating calcium and iodine, are also possible with PC-CT [19]. The virtual non-calcium (VNCa) reconstruction algorithm generates images without calcified lesions while leaving other materials unchanged. This algorithm has been recently investigated by Allmendinger et al. [19] in a phantom study demonstrating good image quality and decreased blooming artifacts when visualizing coronary lumen in the presence of calcification, in comparison with virtual monoenergetic images at an intermediate heart rate. Both UHR acquisition [13, 20] and VNCa reconstruction [19] have been shown to improve the accuracy of stenosis quantification and reduce calcium blooming artifacts; however, it remains unknown which technique should be preferred. Hence, we hypothesized that both VNCa spectral reconstruction and UHR acquisition possible with PC-CT would lead to a similar improvement of stenosis grading compared to conventionally reconstructed images at different heart rates.

The purpose of this study was to assess the impact of using UHR vs VNCa reconstruction on the accuracy of coronary artery stenosis evaluation in comparison to virtual monoenergetic reconstructions acquired at SR with PC-CT. This investigation was conducted using a dynamic motion phantom across varying heart rates.

## Methods

### Phantom

**Fig. 1:**
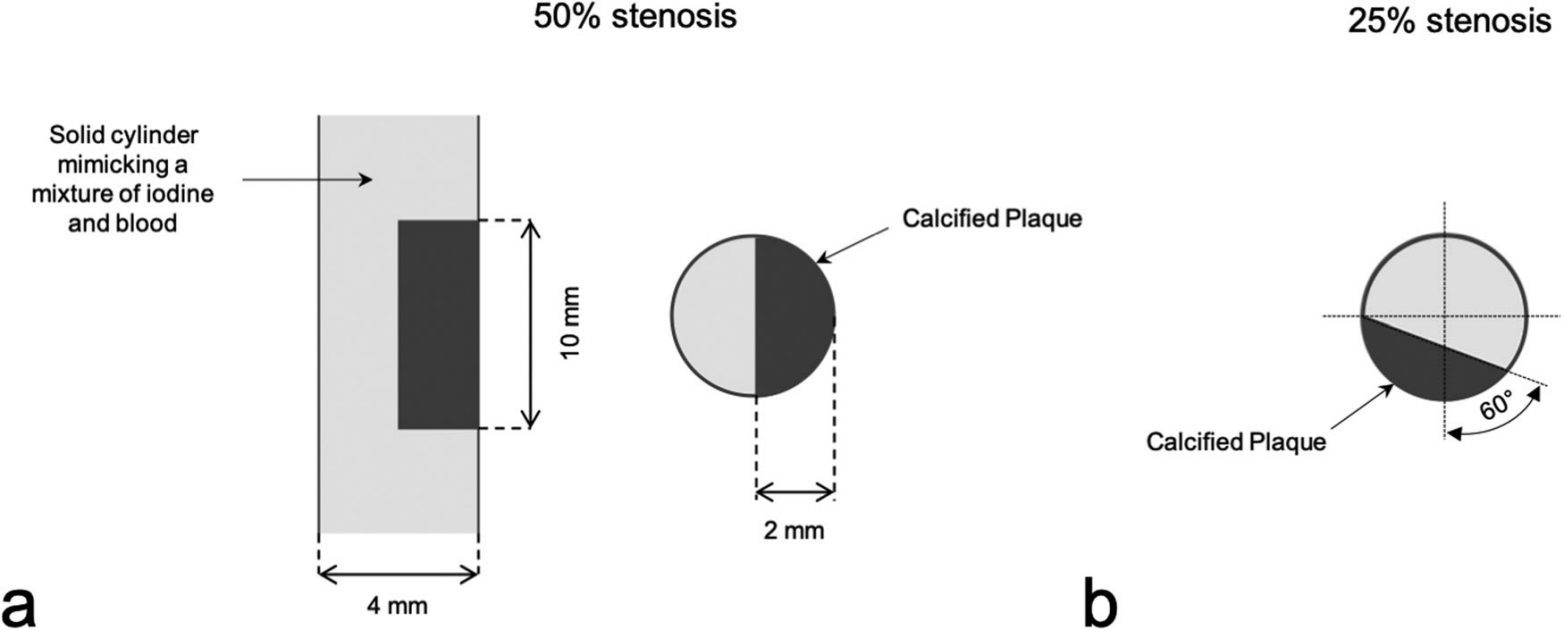
Longitudinal and cross-sectional drawing of 50% stenosis (**a**) and cross-sectional drawing of 25% stenosis (**b**)

A custom-built vessel phantom (Quality Assurance in Radiology and Medicine [QRM], Moehrendorf, Germany), with a 4-mm diameter was used. The vessel was constructed as a solid cylinder simulating a mixture of iodinated contrast material and blood, with a CT value of 800 HU at 120 kVp with conventional CT. Two 10-mm-long calcified lesions composed of hydroxyapatite with a concentration of 800 mg/mL were embedded within the vessel, with a CT value of 1100 HU on conventional CT at 120 kVp. These lesions were placed at various angles with a 5-mm intermittent space designed to induce stenosis of 50% and 25% diameter relative to the vessel diameter. An illustration of the vessel phantom is shown in Fig. 1.

**Fig. 2:**
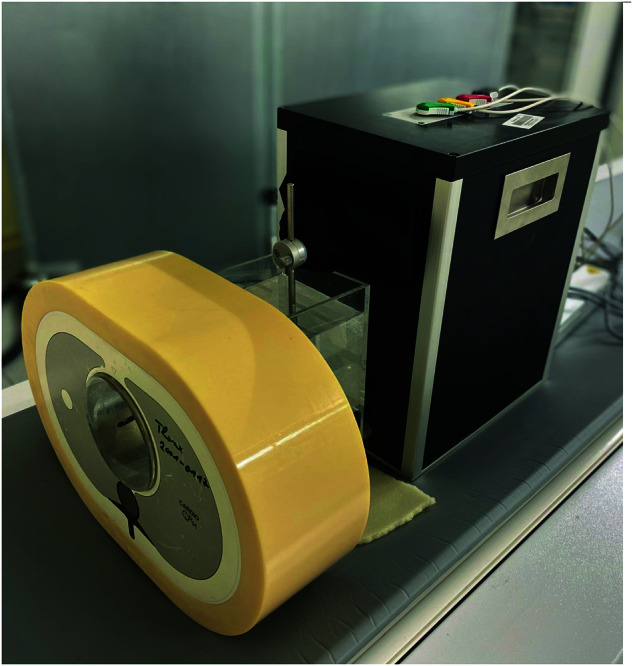
Image capturing the phantom configuration, comprising the chest phantom and the four-dimensional coronary motion simulator

In our study, we used an anthropomorphic chest CT phantom (Cardio CT Phantom, QRM, Moehrendorf, Germany) and a three-dimensional coronary motion simulator phantom (Sim4DCardio, QRM). The thorax phantom, measuring 300 × 200 × 100 mm, reproduced the characteristics of an average-sized patient in terms of thoracic tissue density. Inside the thoracic phantom, a 100-mm diameter water tank housed the coronary motion simulator, which was attached to the vascular phantom. The setup of the phantom is shown in Fig. 2. The coronary motion phantom simulated three-dimensional computer-guided motion along the *x-*, *y-*, and *z*-axes with small amplitudes (2 mm in-plane and 3 mm out-of-plane). This motion phantom generated an artificial electrocardiogram (ECG) signal, simulating heart rates between 50 beats per minute (bpm) and 100 bpm. The motion profiles determined by the manufacturer were derived from clinical coronary vessel velocity profiles originally obtained from electron beam CT data [21].

### Image acquisition and reconstruction

All acquisitions were performed on a whole-body, PC-CT system (NAEOTOM Alpha; Siemens Healthineers, Forchheim, Germany) using an ECG-gated dual-source mode. UHR scans were performed with the following acquisition parameters: tube voltage 120 kVp, collimation 120 × 0.2 mm, gantry rotation time 0.25 s, temporal resolution 66 ms, and ECG pulsing phase at 30–80% of the R-R interval. UHR scans were acquired at heart rates of 60 bpm, 80 bpm, and 100 bpm. In addition to the UHR scans, SR image acquisition was performed with the same parameters, except for a collimation of 144 × 0.4 mm.

At the time of the study, the PC-CT system allows for the collection of ECG-gated spectral information with standard collimation (144 × 0.4 mm), but not with the UHR mode (120 × 0.2 mm). Therefore, virtual monoenergetic images (referred to as SR) and VNCa images were reconstructed from data acquired with standard collimation, while UHR images were reconstructed as a separate set of images (specified as ‘T3D’ by the manufacturer) from data acquired with a collimation of 120 × 0.2 mm.

Reconstructions of phantom data were performed on a dedicated research workstation using proprietary image reconstruction software (ReconCT, version 15.0.58757.0; Siemens Healthineers). UHR images were post-processed using a Bv64 vascular kernel—according to recommendations by Mergen et al. [13]—in the diastolic phase after the selection of % R–R phase with the least motion artifacts, field of view 150 mm, at slice thickness 0.2 mm, increment 0.2 mm, and quantum iterative reconstruction strength level 3, individually for each scan at heart rates of 60 bpm, 80 bpm, and 100 bpm. SR images were reconstructed as traditional monoenergetic images and as VNCa images (PureLumen, Siemens Healthineers). The SR images were reconstructed using virtual monoenergetic images at 55 keV (clinical standard), with the default vascular kernel Bv40, field of view 150 mm, slice thickness 0.6 mm, increment 0.4 mm, and quantum iterative reconstruction strength level 3; these images served as the reference standard. VNCa images were reconstructed using virtual monoenergetic images at 65 keV (vendor recommendation for VNCa), with a Qr44 kernel, while all other reconstruction parameters were matched to those used for the SR images. The VNCa algorithm used in our study is based on spectrally resolved multi-threshold PC-CT data allowing for reconstruction of images without all contributors from calcium or bone-like materials while leaving other material’s attenuation values unchanged. A detailed description of the algorithm can be found in a previous publication by Allmendinger et al. [19].

### Quantitative analysis

**Fig. 3:**
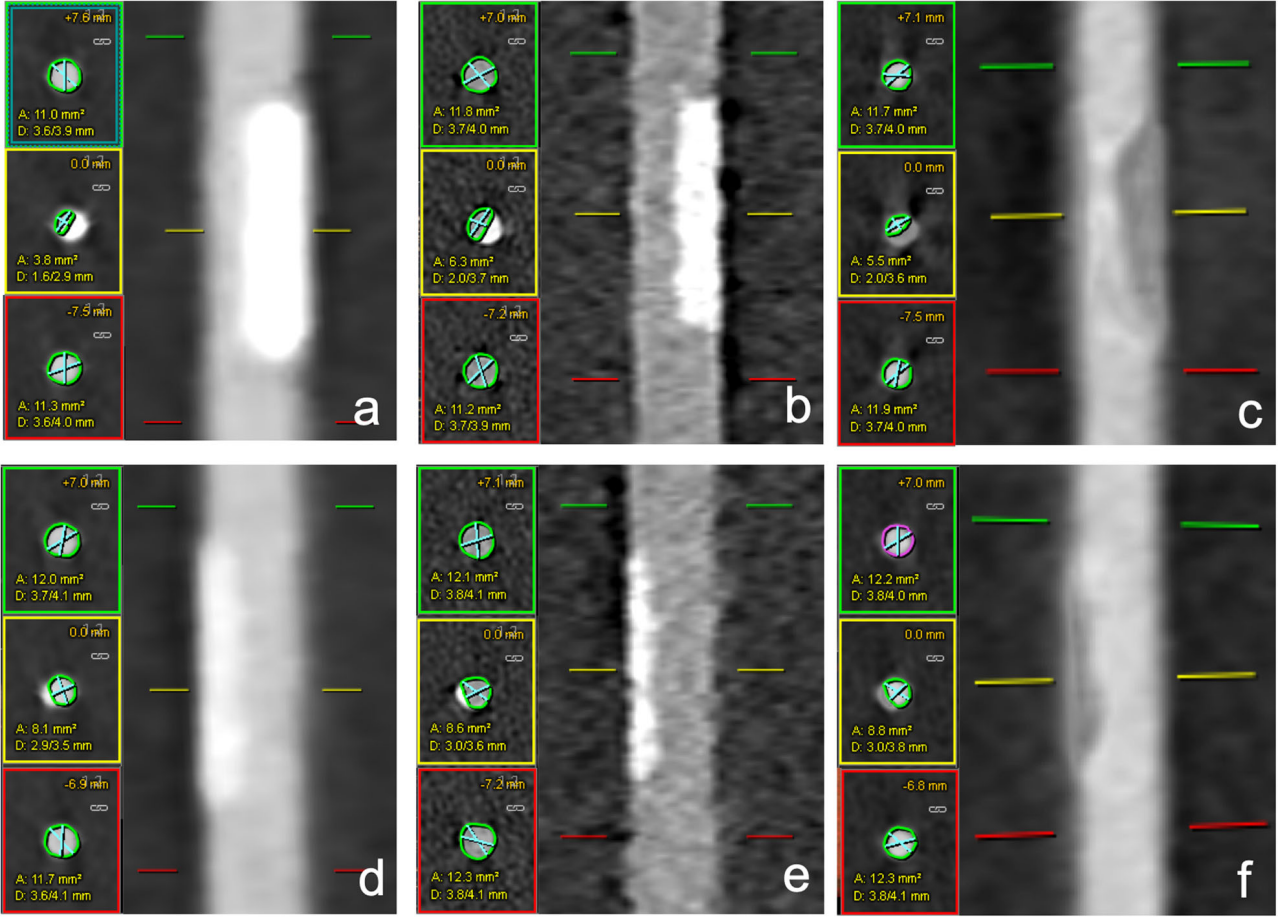
Example of stenosis quantification for the vessel phantom at 60 bpm for the 50% stenosis with SR (**a**), UHR (**b**), VNCa (**c**), and for the 25% stenosis with SR (**d)**, UHR (**e**), and VNCa (**f**) using commercial software (Syngo.via, Siemens Healthineers). Stenosis was identified with markers at the yellow (middle), upper reference at the green (top), and lower reference at the red (bottom) locations. *SR,* standard resolution; *UHR,* ultra-high resolution; *VNCa*, virtual non-calcium

Quantitative analysis was performed with commercially available software (CT Coronary, Syngo.via, Siemens) on the UHR, VNCa, and SR scans as shown in Fig. 3. The percent diameter stenosis (PDS) was calculated as follows [19]:

$${{{{\rm{PDS}}}}}\,=\,\left[1\,-\,\left(\,\frac{{{{{{\rm{D}}}}}}_{{{{{\rm{L}}}}}}\,}{{{{{{\rm{D}}}}}}_{{{{{\rm{V}}}}}}}\right)\right]\times \,100$$

where *D*_L_ represents the minimal lumen diameter measured in cross-sectional images at the site of the lesions, and *D*_v_ is the average of the normal vessel lumen diameters proximal and distal to the stenosis. PDS was measured in percentages at each calcified lesion. Window/level settings were set for each reconstruction to the same values (center 450 HU, width 1500 HU).

Quantitative measurements were performed independently by three readers with 4 years, 2 years, and 2 years of experience in cardiovascular imaging, repeating the measurements three consecutive times.

### Statistical analysis

Statistical analysis was performed in MedCalc Statistical Software Version 19.2.6 (MedCalc Software Ltd.; Ostend, Belgium) and SPSS Version 28.0.1.0 (IBM; Chicago, IL, USA). Continuous data were tested for normality with the Shapiro-Wilk test. PDS values are reported as mean ± standard deviation if normally distributed, median with interquartile range if non-normally distributed.

The differences in PDS_SR_, PDS_UHR,_ and PDS_VNCa_ were assessed by box and whisker plots and paired *t*-test or Wilcoxon test, depending on the distribution. Bonferroni correction was applied and a *p*-value of 0.05/3 = 0.017 was considered significant for testing the three different reconstructions. The agreement was examined both within (intra-reader) and across (inter-reader) readers for all measurements using intraclass correlation coefficient (ICC) with two-way mixed effects and absolute agreement (0.0–0.3, lack of agreement; 0.31–0.5, weak; 0.51–0.7, moderate; 0.71–0.9, strong; and 0.91–1.00, very strong agreement [22]).

## Results

**Fig. 4:**
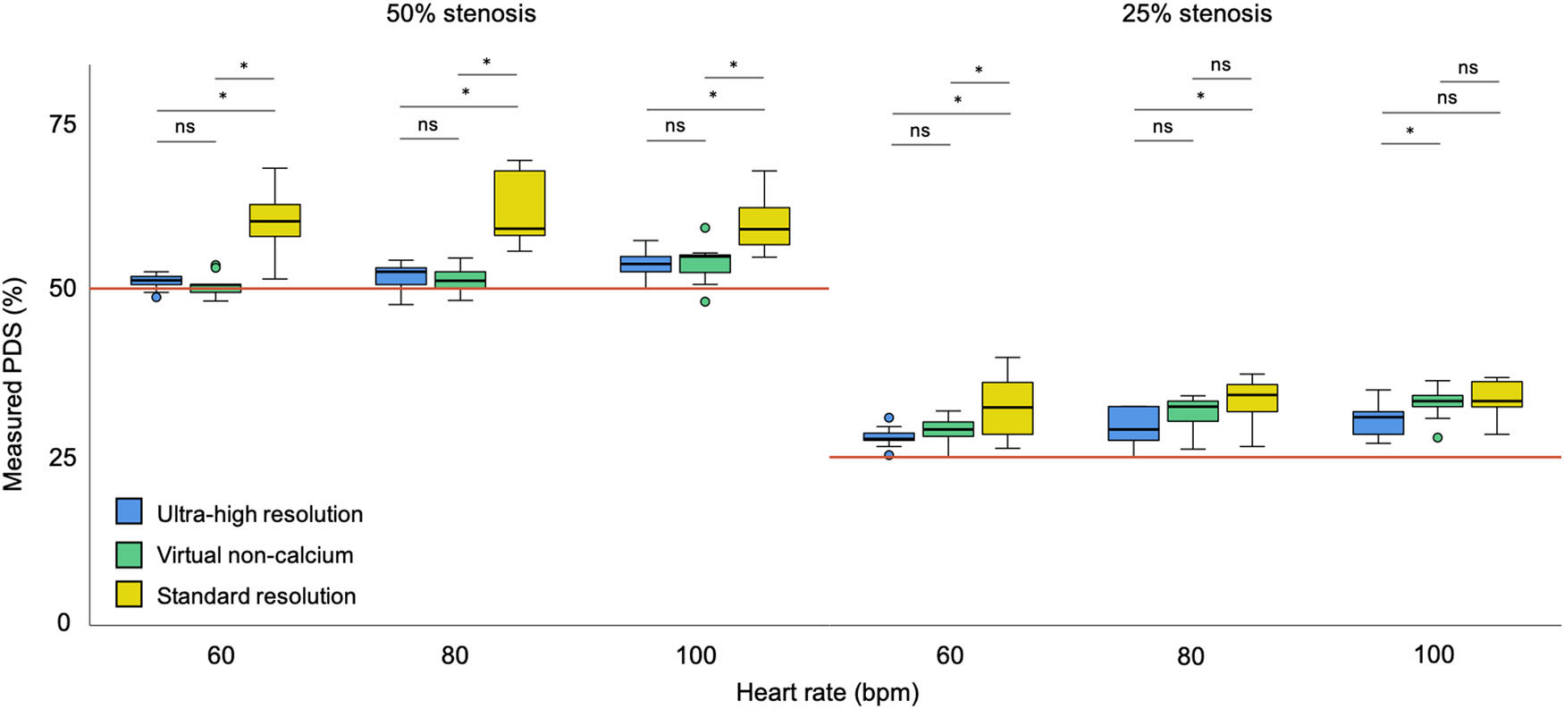
Comparison of measured PDS values illustrated as paired box and whisker plots for the UHR, VNCa, and SR reconstructions at different heart rates. ns, *p* > 0.017; *p ≤ 0.017; **p ≤ 0.001. See the “Methods” section for Bonferroni correction

The measured PDS values with UHR, VNCa, and SR techniques are reported in Tables 1–3 and Fig. 4. For the 50% stenosis, there was a significant difference between PDS_UHR_ compared to PDS_SR_ at all heart rates, and PDS_UHR_ measurements were closer to the nominal stenosis. The comparison of PDS_VNCa_ and PDS_SR_ values also showed a significant difference for the 50% stenosis at all heart rates with PDS_VNCa_ closer to the actual stenosis size. When comparing values between PDS_UHR_ and PDS_VNCa_ for the 50% lesion, there was no significant difference at any heart rate.

**Table 1:** Comparison of PDS values between SR and UHR techniques

	HR (bpm)	PDS_SR_	PDS_UHR_	*p*-value
50% Stenosis	60	60.3 ± 4.9	51.0 ± 1.3	**0.011**
	80	59.6 ± 3.4	51.7 ± 2.2	**0.008**
	100	59.0 ± 2.9	53.7 ± 2.1	**0.011**
	All	59.6 ± 2.6	52.1 ± 2.1	**<** **0.001**
25% Stenosis	60	33.7 ± 3.3	28.0 ± 1.6	**0.008**
	80	34.3 ± 1.9	28.4 ± 2.3	**0.015**
	100	34.0 ± 2.1	29.9 ± 2.6	0.028
	All	33.9 ± 2.5	28.8 ± 2.3	**<** **0.001**
Overall	All	45.6 [33.3–59.0]	41.3 [28.4–52.5]	**<** **0.001**

Values are mean ± standard deviation or median [interquartile range], depending on the distribution. Significant *p*-values after Bonferroni correction in bold characters

*bpm*, beats per minute; PDS, percent diameter stenosis; *SR*, standard resolution; *UHR*, ultra-high resolution

**Table 2:** Comparison of PDS values between SR and VNCa techniques

	Heart rate (bpm)	PDS_SR_	PDS_VNCa_	*p*-value
50% Stenosis	60	60.3 ± 4.9	50.6 ± 1.7	**0.011**
	80	59.6 ± 3.4	51.5 ± 2.1	**0.008**
	100	59.0 ± 2.9	53.7 ± 3.1	**0.011**
	All	59.6 ± 2.6	51.9 ± 2.6	**<** **0.001**
25% Stenosis	60	33.7 ± 3.3	29.1 ± 2.0	**0.008**
	80	34.3 ± 1.9	30.7 ± 2.3	0.110
	100	34.0 ± 2.1	33.1 ± 2.6	0.859
	All	33.9 ± 2.5	30.9 ± 2.8	**<** **0.001**
Overall	All	45.6 [33.3–59.0]	42.5 [30.8–51.2]	**<** **0.001**

Values are mean ± standard deviation or median [interquartile range], depending on the distribution. Significant *p*-values after Bonferroni correction in bold characters

*bpm*, beats per minute; *PDS*, percent diameter stenosis; *SR*, standard resolution; *VNCa*, virtual non-calcium

**Table 3:** PDS values measured by UHR and VNCa techniques

	Heart rate (bpm)	PDS_UHR_	PDS_VNCa_	*p*-value
50% Stenosis	60	51.0 ± 1.3	50.6 ± 1.7	0.327
	80	51.7 ± 2.2	51.5 ± 2.1	0.674
	100	53.7 ± 2.1	53.7 ± 3.1	0.859
	All	52.1 ± 2.1	51.9 ± 2.6	0.619
25% Stenosis	60	28.0 ± 1.6	29.1 ± 2.0	0.260
	80	28.4 ± 2.3	30.7 ± 2.3	0.138
	100	29.9 ± 2.6	33.1 ± 2.6	0.038
	All	28.8 ± 2.3	30.9 ± 2.8	**0.005**
Overall	All	41.3 [28.4–52.5]	42.5 [30.8–51.2]	0.067

Values are mean ± standard deviation or median [interquartile range], depending on the distribution. Significant *p*-values after Bonferroni correction in bold characters

*bpm*, beats per minute; *PDS*, percent diameter stenosis; *UHR*, ultra-high resolution; *VNCa*, virtual non-calcium

For the 25% lesion, there was a significant difference between PDS_UHR_ compared to PDS_SR_ at 60 bpm and 80 bpm, with PDS_UHR_ measurements closer to the actual lesion size, and there was no difference at 100 bpm. When comparing PDS_VNCa_ to PDS_SR_, there was a significant difference at 60 bpm, with PDS_VNCa_ closer to the actual stenosis size, but there was no difference at 80 bpm and 100 bpm. The comparison of PDS_UHR_ and PDS_VNCa_ measurements showed no significant difference at 60 bpm and 80 bpm. However, the difference was significant at 100 bpm, with PDS_UHR_ measurements closer to the actual stenosis size.

The inter-reader agreement was strong for PDS_SR_ (ICC = 0.90, 95% CI: 0.80–0.96), and very strong for PDS_UHR_ (ICC = 0.97, 95% CI: 0.94–0.99) and for PDS_VNCa_ (ICC = 0.95, 95% CI: 0.89–0.98). Intra-reader agreement was very strong for all three readers (Reader 1: ICC = 0.96, 95% CI: 0.91–0.98; Reader 2: ICC = 0.97, 95% CI: 0.93–0.99; Reader 3: ICC = 0.95, 95% CI: 0.90–0.98).

**Fig. 5:**
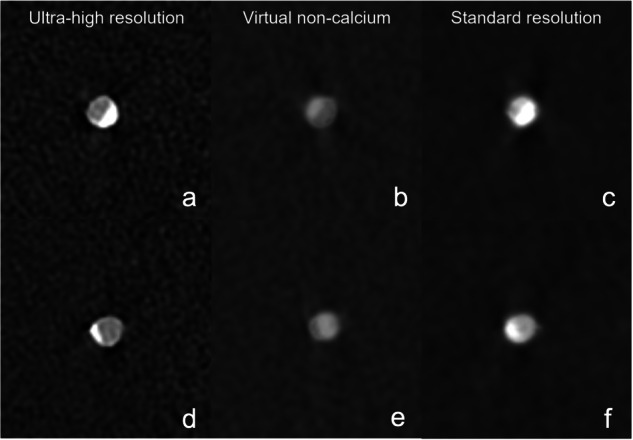
Axial image examples at 60 bpm. Top row shows the 50% lesion with UHR (**a**), VNCa (**b**), and SR (**c**). The bottom row shows the 25% lesion with UHR (**d**), VNCa (**e**), and SR (**f**) techniques

Figure 5 represents sample images of the 50% and 25% lesions at a heart rate of 60 bpm for UHR, VNCa, and SR.

## Discussion

This phantom study evaluated the application of UHR acquisition and VNCa reconstruction in comparison to conventional SR acquisition using a PC-CT system in a coronary artery motion phantom. Our results indicate that both the UHR and the VNCa techniques provide improved quantification for 50% stenosis from low to high heart rates when compared to SR. UHR improved the quantification for the small stenosis as well, independent of heart rate, while VNCa maintained its performance for the 25% stenosis up to 80 bpm.

Our study results show that PDS measurements with UHR and VNCa were closer to the nominal stenosis compared to SR. These results resemble a recent publication by Allmendinger et al. [19] in which the VNCa reconstruction performed well in the presence of motion up to a heart rate of 80 bpm. However, in that study, only qualitative analysis was performed for each heart rate, and quantitative evaluation was implemented only in a pooled fashion. In our study, PDS measurements showed consistently closer values to the nominal stenosis with UHR and VNCa for both lesions at all investigated heart rates. Furthermore, all three reconstructions showed strong to very strong ICC, however, UHR and VNCa further improved the inter-reader agreement when compared to SR. Reproducibility was assessed with intra-reader agreement analysis which showed very strong agreement for all three readers. However, it is worth mentioning that our measurements at SR for the 50% stenosis did not show a consistent increase with increasing heart rates. Similar results were reported by a recent study [23] investigating the stability of spectral results at different heart rates, acquisition modes, and cardiac phases using the same CT system and motion phantom. The authors did not demonstrate a consistent increase in spectral results with increasing heart rates at diastole with virtual monoenergetic reconstructions.

While coronary CT angiography is considered a first-line noninvasive test for the evaluation and management of coronary artery disease [3, 24], stenosis quantification remains a challenge in the presence of severe calcifications mostly due to blooming artifacts. Recent advances, such as UHR acquisition and VNCa reconstruction using PC-CT, however, may address this shortcoming by improving the visualization of coronary plaques and adjacent vessel lumen. Clinically, this has the potential to improve the specific and positive predictive value of coronary CT angiography.

Recent patient and phantom studies investigated prototype and clinical UHR PC-CT (slice thickness of 0.2 mm, 0.25 mm, and 0.275 mm) for coronary imaging and showed promising results over SR PC-CT (0.6 mm) with improved visualization of coronary plaques and stents [25–29]. The feasibility and image quality of the current clinical UHR PC-CT for coronary imaging was first investigated by Mergen et al. [30] in which they determined the optimal reconstruction kernel for patients with high coronary calcium load. They suggested the use of Bv64 as the best kernel for plaque characterization, blooming artifact reduction, and the delineation of coronary artery lumen with UHR [31]. They demonstrated reduced blooming artifacts, high image quality, and high vessel sharpness at UHR (slice thickness of 0.2 mm) in comparison to images reconstructed at SR (0.6 mm). The major limitation of the Mergen et al. [31] study is the lack of a reference standard for stenosis measurement, as invasive coronary angiography was not performed in their investigation. Our study addresses such limitations by having ground truth measurements available as a reference for stenosis quantification. In addition, Koons et al. [32] evaluated the potential of UHR PC-CT in quantifying one-sided and ring-shaped stenosis in comparison to conventional CT using a static vessel phantom. PC-CT was found to be more accurate than conventional CT for all phantom configurations, especially for ring-shaped plaques. While the aforementioned papers demonstrated the feasibility of PC-CT at UHR for plaque visualization and quantification, its potential for improving stenosis quantification over various heart rates has not been explored.

VNCa algorithm, a possible alternative technique to UHR acquisition with the potential to improve stenosis quantification, has been shown to improve image quality and decrease blooming artifacts in vessel phantoms [19].

As demonstrated in our study, both UHR and VNCa have benefits over SR acquisition for coronary artery stenosis quantification, even at higher heart rates. From a clinical perspective, this holds significant merit, especially in cases where the patient presents for coronary CT angiography with an elevated heart rate, and the administration of beta-blockers is deemed contraindicated. Moreover, the expeditious performance of CT scans becomes advantageous if beta-blocker intervention is reserved exclusively for individuals manifesting markedly elevated heart rates surpassing 80 bpm, facilitating faster patient care. However, the current UHR PC-CT technique has its shortcomings, e.g., the lack of spectral acquisition at UHR, meaning that VNCa reconstruction cannot be performed on UHR data due to current technical limitations. While the SR spectral mode allows for a high pitch acquisition, this is unavailable with UHR acquisition due to the current purposefully limited *z*-axis coverage. As a consequence, sequential or spiral mode needs to be used to obtain a full image using UHR, leading to either higher radiation doses or requiring more steps and longer acquisition time, than by using the spectral mode with standard collimation. However, our present results suggest that the use of SR with VNCa may provide a solution to this. In a clinical setting, there may be patients who would benefit from either VNCa at SR or standard reconstruction at UHR. The decision between the two techniques would likely depend on the heart rate (as UHR performed better > 80 bpm) and age (out of concern of higher radiation dose when using UHR). We posit that an ideal solution would eventually be the combination of UHR acquisition and VNCa reconstruction, which may potentially omit blooming artifacts and improve stenosis quantification to the highest level, further improving the positive predictive value of coronary CT angiography in the diagnosis of coronary artery disease.

Our study has some limitations. First, since this was a phantom experiment, the clinical implications of our results warrant further investigations. Second, different vessel sizes and contrast material concentrations were not evaluated and the vessel phantom used had a diameter of 4 mm, which is usually the diameter of the left main coronary artery. The other coronary arteries usually have a diameter of less than 4 mm, therefore it should be investigated whether our findings are also true for vessels with smaller sizes. Third, only calcified plaques with a given density were available. As a consequence, further plaque types were not evaluated. Fourth, the lesions in the vessel phantom had a clear-cut shape and the size distribution was restricted to 50% and 25%. Thus, further studies should evaluate high-grade and irregularly shaped stenoses. Fifth, only one reconstruction setting was used for image postprocessing. Therefore future research should determine the influence of different reconstruction parameters on the performance of VNCa and UHR acquisition-based stenosis grading. Finally, to enable the widespread implementation of these techniques in clinical practice, future studies are warranted to assess the feasibility of the VNCa algorithm in patients and to provide recommendations in which cases UHR vs VNCa should be used when quantifying coronary artery stenosis.

In conclusion, this motion phantom study demonstrated improved stenosis quantification accuracy with PC-CT using either UHR acquisition or VNCa reconstruction techniques even at high heart rates compared to SR.

## Data Availability

The datasets used and/or analyzed during the current study are available from the corresponding author on reasonable request.

## Abbreviations

bpm: Beats per minute
CT: Computed tomography
ECG: Electrocardiogram
ICC: Intraclass correlation coefficient
PC-CT: Photon-counting CT
PDS: Percent diameter stenosis
SR: Standard resolution
UHR: Ultra-high resolution
VNCa: Virtual non-calcium
